# High-Dimensional Single-Cell Quantitative Profiling of Skeletal Muscle Cell Population Dynamics during Regeneration

**DOI:** 10.3390/cells9071723

**Published:** 2020-07-18

**Authors:** Lucia Lisa Petrilli, Filomena Spada, Alessandro Palma, Alessio Reggio, Marco Rosina, Cesare Gargioli, Luisa Castagnoli, Claudia Fuoco, Gianni Cesareni

**Affiliations:** 1 Department of Biology, University of Rome “Tor Vergata”, 00133 Rome, Italy; lucialisa.petrilli@opbg.net (L.L.P.); filomena.spada86@gmail.com (F.S.); alessandro.palma@live.it (A.P.); alessio.reggio@uniroma2.it (A.R.); marco.rosina90@gmail.com (M.R.); Cesare.Gargioli@uniroma2.it (C.G.); castagnoli@uniroma2.it (L.C.); cesareni@uniroma2.it (G.C.); 2 Department of Onco-hematology, Gene and Cell Therapy—Bambino Gesù Children’s Hospital—IRCCS, 00146 Rome, Italy; 3 Fondazione Santa Lucia Istituto di Ricovero e Cura a Carattere Scientifico (IRCCS), 00143 Rome, Italy

**Keywords:** single-cell, mass cytometry, skeletal muscle regeneration, skeletal muscle homeostasis, fibro/adipogenic progenitors, myogenic progenitors, muscle populations

## Abstract

The interstitial space surrounding the skeletal muscle fibers is populated by a variety of mononuclear cell types. Upon acute or chronic insult, these cell populations become activated and initiate finely-orchestrated crosstalk that promotes myofiber repair and regeneration. Mass cytometry is a powerful and highly multiplexed technique for profiling single-cells. Herein, it was used to dissect the dynamics of cell populations in the skeletal muscle in physiological and pathological conditions. Here, we characterized an antibody panel that could be used to identify most of the cell populations in the muscle interstitial space. By exploiting the mass cytometry resolution, we provided a comprehensive picture of the dynamics of the major cell populations that sensed and responded to acute damage in wild type mice and in a mouse model of Duchenne muscular dystrophy. In addition, we revealed the intrinsic heterogeneity of many of these cell populations.

## 1. Introduction

In physiological conditions, the adult skeletal muscle has a relatively low cell turnover [1]. However, physical activity, trauma, or muscle pathologies, undermining tissue integrity, trigger a tightly controlled regeneration process. Although satellite cells (SCs) are the main actors of myofiber regeneration after damage [2,3,4,5,6], successful muscle healing requires the participation of additional cell types that directly or indirectly contribute to this process. In this context, immune cells and fibro/adipogenic progenitors (FAPs) play a prominent role in supporting the clearance of the damaged tissue, while assisting SCs in their regenerative role [7,8,9,10,11]. However, the orchestrated crosstalk of the regeneration machinery gradually fails in patients affected by muscle-related disorders, such as dystrophies [12]. Here, the accumulation of intrinsic cell defects and the changes in the stem cell niche lead to infiltrations of fat and fibrotic deposition, compromising muscle functions [9,13,14,15,16].

Over the past decades, the complex cell crosstalk occurring during muscle regeneration has been studied in detail [17,18,19]. However, to date, most studies have mainly relied on the analysis of bulk cell populations identified by the expression of a few specific markers and sorted for ex vivo analysis. As a consequence, due to the lack of technologies suitable to address this issue, little is known about muscle cell population heterogeneity. Only recently, the development of technologies to determine the transcriptome of single-cells or their exposed antigen repertoires has permitted to reveal the extent of this heterogeneity and its possible implication in muscle physiology and pathology [20,21,22,23,24]. Moreover, the dynamic changes and the relative abundance of muscle cell populations, upon acute or chronic damage, still remain largely uncharacterized. To fill this gap, we explored, via single-cell mass cytometry, the changes in the multidimensional antigen repertoires of the main players, colonizing the muscle stem cell niche after injury. Here, we described, at single-cell resolution, the time-dependent changes of muscle population dynamics upon myotoxin-induced damage in wild type (wt) and in a mouse model of Duchenne muscular dystrophy (the mdx model).

## 2. Materials and Methods

### 2.1. Mouse Strains and Animal Procedures 

C57BL/6J (RRID:IMSR_JAX:000664) and C57BL/10ScSn-Dmd^mdx^/J mice (RRID:IMSR_JAX:001801), hereafter referred to as wt and mdx mice, respectively, were purchased from the Jackson Laboratory.

Mice were bred respecting the standard animal facility procedures, and all the procedures were conducted in accordance with rules of good animal experimentation I.A.C.U.C. n°432 of 12 March 2006 and under ethical approval released on 23/October/2017 from the Italian Ministry of Health, protocol #820/2017-PR. 

For muscle injury, 45-day-old wt and mdx mice were anesthetized with an intramuscular injection of saline solution containing ketamine (5 mg/mL) and xylazine (1 mg/mL) prior to the intramuscular administration of 20 μL of 10 μM cardiotoxin solution, isolated from *Naja Pallida* (Latoxan L8102, Portes les valence, France), into *tibialis anterior*, *quadriceps,* and *gastrocnemius* muscles. 

### 2.2. Histological Analysis 

*Tibialis anterior* (TA) muscles were collected, embedded in optimal cutting temperature compound (Killik—O.C.T., Bio Optica, Milan, Italy), and snap-frozen in liquid nitrogen for 10 s. Embedded muscles were stored at −80 °C for transverse cryo-sectioning with a Leica cryostat. Cryosections (10 μm thickness) were collected on Superfrost glass slides (Thermo Fisher Scientific, Monza, Italy), and tissue slides were stained with hematoxylin and eosin (H&E).

For the H&E, cryosections were fixed with 4% paraformaldehyde (PFA, Santa Cruz Biotechnology, D.B.A. Italia S.r.l., Segrate Milan, Italy) for 15 min at room temperature (RT). After washing in 1X PBS, tissue slides were incubated in the hematoxylin solution for 15 min and rinsed for 5 min in tap water. Cryosections were then counterstained with an alcoholic solution of eosin for 30 min. Following the eosin staining, cryosections were dehydrated in increasing concentrations of alcohol, clarified with the histo-clear solution (Agar Scientific Ltd, Stansted, UK), and finally mounted on coverslips, using the resinous Eukitt mounting medium (Electron Microscopy Sciences, Hatfield Township, PA, USA).

H&E images were captured using the Zeiss Lab A1 AX10 microscope at the 20× magnification in the bright field.

### 2.3. Skeletal Muscle Mononuclear Cell Purification

Isolation of mononuclear cell populations was performed as in Spada et al. [25]. Mice were sacrificed by cervical dislocation, and the hind limbs were washed with 70% ethanol. Mice hind limbs were then dissected and finely minced in Hank’s balanced salt solution (HBSS) with calcium and magnesium (Gibco- Thermo Fisher Scientific, Monza, Italy) supplemented with 0.2% bovine serum albumin (BSA) (AppliChem, Cinisello Balsamo, Milan, Italy) and 1% penicillin-streptomycin (P/S) (Life Technologies, Monza, Italy, 10,000 U/mL) (HBSS^+^) under a sterile hood. The homogenized tissue preparation was centrifuged at 70× *g* for 10 min at 4 °C to separate fat and subjected to enzymatic digestion for 1 h at 37 °C, with gentle mixing in a solution containing 2 μg/μL collagenase A (Roche- Merck KGaA, Darmstadt, Germany), 2.4 U/mL dispase II (Roche- Merck KGaA, Darmstadt, Germany), and 10 μg/mL DNase I (Roche- Merck KGaA, Darmstadt, Germany) diluted in Dulbecco’s phosphate-buffered saline (D-PBS) with calcium and magnesium (Gibco-Thermo Fisher Scientific, Monza, Italy). The reaction was inactivated with HBSS^+^, and the cell suspension was subjected to three sequential filtrations through 100 μm, 70 μm, and 40 μm cell strainers (BD Falcon, BD Italia, Milan, Italy) and centrifugations at 700× *g* for 5 min. The lysis of red blood cells was performed by incubating with RBC Lysis Buffer (Santa Cruz Biotechnology, D.B.A. Italia S.r.l., Segrate, Milan, Italy) for 150 s on ice, prior to the 40 µm filtration step.

### 2.4. Single-Cell Mass Cytometry

For single-cell mass cytometry experiments, 3 × 10^6^ cells were used for each condition. Each time point was analyzed in triplicate, starting from mononuclear cells purified from three different mice. Cells were centrifuged at 600× *g* for 5 min and washed in D-PBS w/o calcium and magnesium (BioWest- VWR INTERNATIONAL PBI S.r.l., Milan, Italy). To minimize the inter-sample antibody staining variation, we applied a mass-tag barcoding protocol on fixed cells. Cells were fixed with 1 mL of Fix I Buffer (Fluidigm, South San Francisco, CA, USA) and then incubated for 10 min at RT. The fixation was quenched with Barcode Perm Buffer (Fluidigm, South San Francisco, CA, USA). The different samples were barcoded by individually incubating the cell suspensions with the appropriate combination of palladium isotopes from the Cell-ID^TM^ 20-Plex Pd Barcoding Kit (Fluidigm, South San Francisco, CA, USA) in Barcode Perm Buffer for 30 min at RT. The staining was quenched with MaxPar Cell Staining Buffer (Fluidigm, South San Francisco, CA, USA).

The antibody staining with metal-tagged antibodies that target surface and intracellular antigens was performed on the samples pooled after mass-tag barcoding. Samples were collected in a single tube, and the surface antibody staining protocol was performed according to manufacturers’ instructions for 30 min at RT. Surface-stained cells were then washed twice with MaxPar Cell Staining Buffer (Fluidigm South San Francisco, CA, USA) and permeabilized with ice-cold methanol for 10 min on ice. Membrane-permeabilized cells were washed twice with MaxPar Cell Staining Buffer (Fluidigm, South San Francisco, CA, USA) and incubated with antibodies against intracellular antigens for 30 min at RT according to manufacturers’ instructions. The full list of antibodies is detailed in Table 1. All the antibodies listed were purchased from Fluidigm (South San Francisco, CA, USA). After intracellular antibody staining, cells were washed twice with MaxPar Cell Staining Buffer and stained for 1 h at RT with the intercalation solution, composed of Cell-ID Intercalator-Ir (191Ir and 193Ir, Fluidigm South San Francisco, CA, USA) in MaxPar Fix and Perm Buffer (Fluidigm, South San Francisco, CA, USA) at a final concentration of 125 nM. Cells were washed twice with MaxPar Cell Staining Buffer and MaxPar Water.

For mass cytometry analysis, cells were resuspended at the final concentration of 2.5 × 10^5^ cells/mL in MaxPar Water containing 10% of EQ^TM^ Four Element Calibration Beads (Fluidigm, South San Francisco, CA, USA) and filtered through a 30-μm filter-cap FACS tube. Samples were kept on ice prior to the acquisition by using the mass cytometry platform CyTOF2 System (Fluidigm, South San Francisco, CA, USA).

### 2.5. CyTOF Data Analysis

Following data acquisition, channel intensity was normalized using calibration beads [26], and the normalized *fcs* file was de-barcoded by using the Debarcoder software (Fluidigm, South San Francisco, CA, USA). Data have been pre-processed using the Cytobank software platform [27]. Cells were manually gated from debris on the basis of DNA content monitored by the incorporation of the iridium (Ir) intercalator. Doublets were then excluded according to the event length parameter, and single live cells were finally manually gated by using the cisplatin (Pt) intercalator signal. Manually gated singlet (191Ir^+^ 193Ir^+^), viable (195Pt^−^) events were imported into Cytofkit for further analysis [28]. Cytofkit [28] parameters were set as follows: 14 biomarkers were included for clustering all the detected live cells per sample (“all” merge method); transformation method: cytofAsinh; FlowSOM was used as clustering algorithm with *k = 15*, tSNE perplexity set to 30; 2000 iterations and seed: 42.

Data were analyzed with Cytofkit shiny app [28] https://github.com/JinmiaoChenLab/cytofkit) and R scripts. Gating for marker-positive cells was performed by setting the mean +/- standard deviation as a threshold, depending on the expression value distribution of the specific marker.

### 2.6. Statistical Analysis 

The experiments were performed at least in biological triplicates, that is, from at least 3 independent mononuclear cell preparations for each experiment. Only for the time point at day 5 in the wild type time series, we had just two biological repeats. Results were presented as mean ± SEM unless otherwise mentioned. Statistical evaluation was done by using One-way or Two-way ANOVA. Comparisons were considered statistically significant at * *p* < 0.05; ** *p* < 0.01; *** *p* < 0.001; **** *p* < 0.0001. All statistical analysis was performed using Prism 6 (GraphPad, San Diego, CA, USA). 

## 3. Results

### 3.1. Histological Profiling of Skeletal Muscle Tissue from wt and mdx Muscles Following Acute Damage

**Figure 1:**
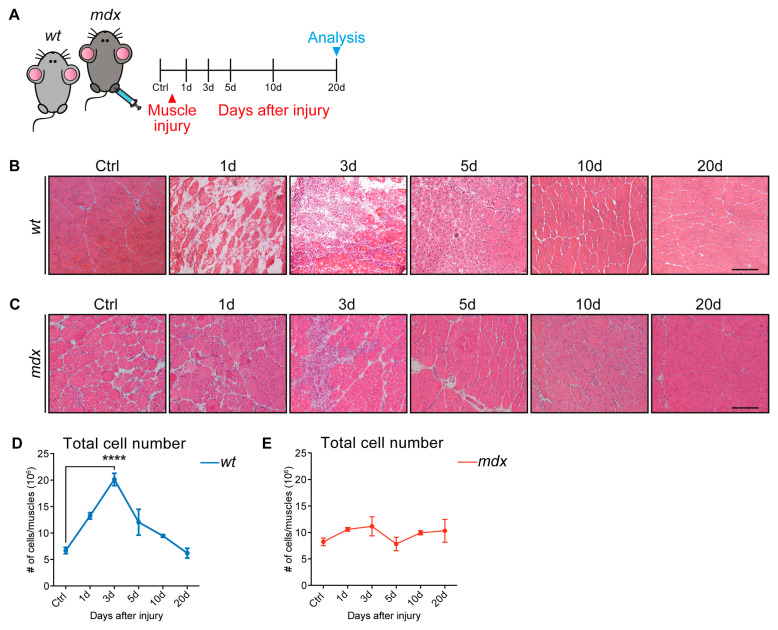
Cardiotoxin-induced injury on wild type (wt) and a mouse model of Duchenne muscular dystrophy (mdx) skeletal muscle tissue. (**A**) Experimental procedure. 45-day-old wt and mdx mice were injected intramuscularly with cardiotoxin (CTX) (10 μM), and the skeletal muscles were analyzed 1, 3, 5, 10, and 20 days (d) after injury. (**B**) Representative hematoxylin and eosin staining of uninjured wt *tibialis anterior* (TA) muscles and regenerating wt TA muscles at 1, 3, 5, 10, and 20 days after intramuscular CTX injection. Regenerating muscles were characterized by centrally located nuclei at day 5, but reconstituted multinucleated myofibers by day 10. (**C**) Representative hematoxylin and eosin staining on histological sections of uninjured and regenerating mdx TA muscles at 1, 3, 5, 10, and 20 days after intramuscular CTX injection. All along the considered time points, mdx muscles were characterized by infiltrating inflammatory interstitial cells and centrally nucleated myofibers of different sizes. (**D**,**E**) The number of cells (in millions) extracted from uninjured and CTX-injured wt (**D**) and mdx (**E**) mice (n=3; for 5d wt time point, n=2). All data were represented as mean ± SEM, and the statistical significance was estimated by two-way ANOVA (**** *p* < 0.0001). (**B**,**C**) 20× magnification; scale bar: 100 μm.

To gain insights into the skeletal muscle repair process, we aimed at describing the dynamics of muscle cell populations following acute damage. To induce muscle injury, we used a well-established protocol based on the injection of the snake (*Naja pallida*) myotoxin (cardiotoxin, CTX) into the hind limb muscles of wt and mdx dystrophic mice (Figure 1A) [29,30]. Cardiotoxin, by inhibiting protein kinase C (PKC), induced the increase of cytosolic calcium, causing myofiber myolysis that, in turn, triggers regeneration [31,32,33]. First, we monitored, by hematoxylin and eosin staining, the changes in the skeletal muscle architecture at five different time points that were chosen to monitor the key events of the muscle healing process after damage: necrosis, inflammation, regeneration, and remodeling (Figure 1B,C).

Sections of uninjured wt muscles were characterized by polygonal fibers of uniform size containing peripheral nuclei (Figure 1B) [34]. Upon CTX injection, the skeletal muscle underwent degeneration, setting in motion the regeneration process. The degeneration of the muscle architecture was clearly observable at day 1 after injury, while interstitial cells, either resident cell populations [8] or infiltrating immune cells [35], became conspicuous at day 3 after damage. The regeneration process was completed after 20 days, as highlighted by the presence of multinucleated regenerated myofibers.

The mdx skeletal muscle, on the other hand, even in the absence of acute insult, was characterized by infiltrating inflammatory interstitial cells and centrally nucleated myofibers of different sizes, hallmarks of the dystrophic pathology (Figure 1C) [12,36,37]. Following CTX injection, the injured tissue underwent a regeneration process, without being apparently impacted further by extensive necrosis. If anything, the mdx muscle seemed to be more resilient to the myotoxin-induced damage and did not undergo the massive structural damage that was observed early after cardiotoxin injection in the wt muscle. Altogether, the histological analysis showed that the muscles from the two genetic backgrounds responded differently to CTX-induced injury.

### 3.2. Single-Cell Quantitative Profiling of Skeletal Muscle Populations Following Myotoxin-Induced Injury 

Next, we sought to characterize the different mononuclear cell populations in the two mouse models and monitor their abundance changes during the regeneration process. To this end, we resorted to using mass cytometry [8].

In wt muscles, the number of isolated mononuclear cells increased after damage and peaked at day 3 to return to almost baseline levels at day 10 (Figure 1D). In contrast, consistent with the resilience of the mdx muscle observed in the histological analysis, this response was not detected in the injured muscles of mdx mice, where the mononuclear cell number remained constant over the whole regeneration process (Figure 1E).

The mononuclear cell samples from both animal models were separately barcoded and labeled with a panel of 23 metal-tagged antibodies (Table 1) targeting antigens expressed by muscle resident cells and/or by cell populations from the hematopoietic compartment. Mononuclear cells were purified from the uninjured and injured muscles at five time-points and, after barcoding and labeling, analyzed with a CyTOF2 mass cytometer in a single-run experiment [38]. Signals were debarcoded, and live cells were identified using the cisplatin (Pt) intercalator signal. Live/dead cell analysis highlighted that dead wt cells significantly increased at day 3 (Figure S1A), while for mdx muscles, the live/dead cell ratio remained constant (Figure S1B). 

Single-cell data were analyzed by applying, as a dimensionality reduction method, the t-distributed stochastic neighbor embedding (t-SNE) algorithm implemented in Cytofkit [28,39].

**Figure 2:**
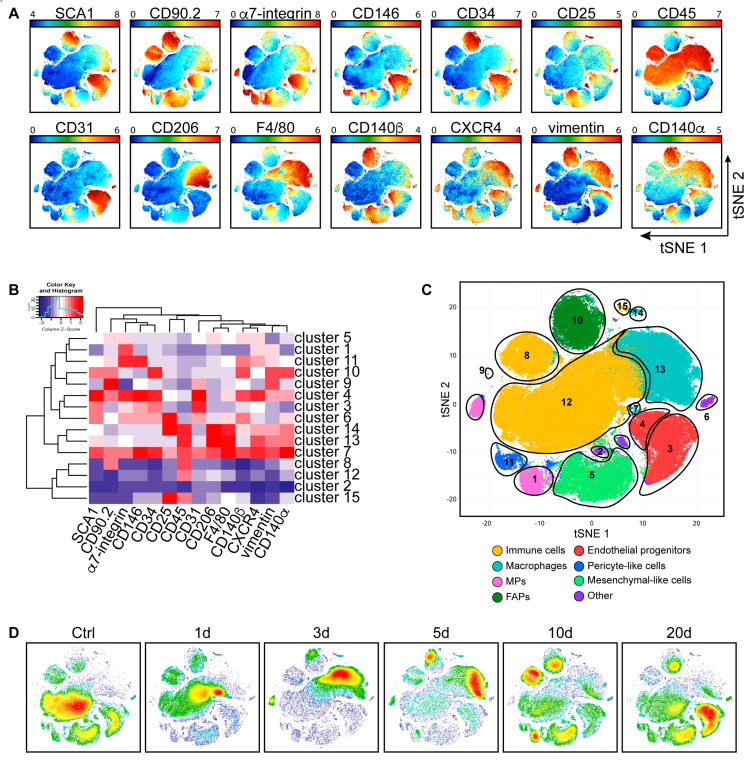
Dynamic changes of mononuclear cell subpopulations in wt muscles during regeneration. (**A**) The different mononuclear cell samples from uninjured and CTX-injured wt hind limb muscles were merged to create a single t-distributed stochastic neighbor embedding (t-SNE) map; colored according to expression levels of SCA1, CD90.2, α7-integrin, CD146, CD34, CD25, CD45, CD31, CD206, F4/80, CD140β, CXCR4, vimentin, and CD140α (blue: low expression; red: high expression of the selected marker). (**B**) FlowSOM heatmap of column normalized (Z-score) marker expression for each of the 15 identified clusters. Colors varied according to the expression level of the considered marker in a blue to red scale, indicating low to high expression, respectively. (**C**) Cell clusters defined by the FlowSOM analysis were assigned to arbitrary colors (yellow: immune cells; light blue: macrophages; pink: myogenic progenitors (MPs); dark green: fibro/adipogenic progenitors (FAPs); red: endothelial progenitors; blue: pericyte-like cells; light green: mesenchymal-like cells; purple: other). (**D**) Time course of the variation in cell subpopulation abundance upon CTX-induced injury. The dynamic changes were illustrated by density plots, colored according to cell density (blue: low density; red: high density).

As the readouts of phospho-antibodies were barely above the background signal, albeit cell-specific (Figure S2), they were not considered in this analysis. The readouts of the 14 antigens in Figure 2A were used as input for the t-SNE algorithm. This approach yielded a two-dimensional map of the antigenic expression profiles of mononuclear cell populations in the wt skeletal muscle (Figure 2A) and led to the identification of 15 different cell clusters (Figure 2B).

The 15 identified clusters (Figure 2B) were further grouped into eight cell types by matching their expression profile to that of cell types already described in the literature [20,40]. More specifically, we were able to identify populations expressing antigens typical of immune cells (CD45^+^) (clusters 7, 8, 12, 13, 14, and 15), macrophages (CD45^+^ and F4/80^+^) (clusters 7, 13, and 14), myogenic progenitors (MPs) (α7-integrin^+^) (cluster 1), fibro/adipogenic progenitors (FAPs) (SCA1^+^, CD34^+^, CD140α^+^, CD90.2^+^, and vimentin^+^) (cluster 10), endothelial progenitor cells (CD31^+^) (clusters 3 and 4), pericyte-like cells (CD146^+^ and CD140β^+^) (cluster 11), and mesenchymal-like cells (CD90.2^+^) (cluster 5). The expression profiles of three remaining non-abundant clusters (clusters 2, 6, and 9) could not be matched to any of the already described muscle cell types and were collectively dubbed “others” (Figure 2B,C).

As we analyzed samples at different time points, we could also monitor the dynamic of cell populations after CTX damage. The picture in Figure 2C, in fact, was not static as the relative abundance of the different cell populations changed along the regeneration process. These could be best appreciated by comparing the bidimensional t-SNE maps at different time points (Figure 2D). Here the colors, relating to cell density, from blue (low density) to red (high density), allowed to appreciate the significant changes in the cell population proportions during regeneration.

To quantitatively describe the dynamics of these changes, we first classified the observed cell populations into two main clusters according to CD45 expression: (i) the immune (CD45^+^) and the (ii) non-immune (CD45^−^) subpopulations of mononuclear cells. The data were shown as population relative abundance in the mononuclear cell preparations analyzed in the CyTOF. However, this data representation could be easily transformed into changes in the absolute numbers of each cell population in the mouse muscle as the total number of mononuclear cells in each condition was known (Figures S3 and S4). The curve trends in the two representations were similar.

**Figure 3:**
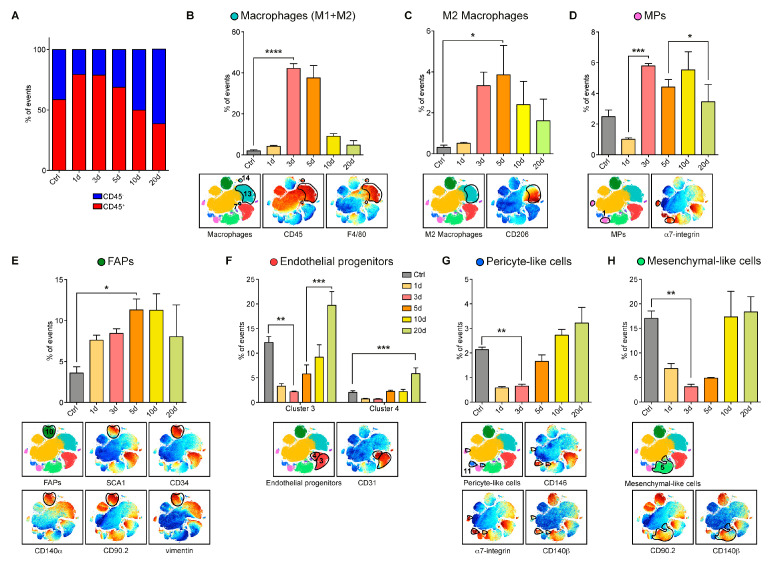
Characterization of the cell-population density rearrangements induced by CTX in the wt skeletal muscle after 1, 3, 5, 10, 20 days. (**A**) Bar plots, showing the CD45^-^ (blue) / CD45^+^ (red) ratio in mononuclear cells from wt skeletal muscles at different time points after CTX injury. Data were represented as mean, while the statistical significance was estimated by two-way ANOVA. (**B–H**) Identification in the t-SNE maps of mononuclear cell populations. The different plots were color-coded according to the expression of surface antigens that characterize the relevant cell types. The bar plots quantitated the variation in population abundance in the wt limb muscles at different times during regeneration. Cell percentages were assessed on the total number of cells in each sample (n = 3; for 5d wt time point, n = 2). The statistical significance was estimated by one-way ANOVA. All data were represented as mean ± SEM, and the statistical significance was defined as * *p* < 0.05; ** *p* < 0.01; *** *p* < 0.001; **** *p* < 0.0001.

We first focused on CD45^+^ hematopoietic cells as they play a critical role in the regeneration process by sending regulatory signals by removing the damaged-fiber debris and by stimulating proliferation and differentiation of myogenic progenitors [19,41,42,43,44,45]. In a homeostatic muscle, approximately 60% of the mononuclear cells exposed the CD45 antigen. This cell compartment increased in number, after damage, to peak at days 1 and 3 (Figure 3A).

In physiological conditions, a fraction of the hematopoietic cells, less than 2% of the total recorded events, also expressed the F4/80^+^ antigen, a pan-macrophage marker (Figure 3B). However, already 3 days after injury, the macrophage population significantly increased, reaching a maximum of approximately 40%. At later times, macrophage abundance gradually decreased, returning close to baseline levels at day 20. The macrophage population is not homogeneous as it contains pro-inflammatory (M1) and anti-inflammatory (M2) macrophages that differ in the expression of the CD206 antigen and play a different role in muscle regeneration [46,47].

Noteworthy, CD206^+^ M2 macrophages, which are responsible for the resolution of the inflammatory response [48,49,50], increased significantly on day 5 at the expense of inflammatory macrophages M1 that peaked at day 3 (Figure 3B,C).

We next focused on the cell populations that did not express the CD45 antigen. This group included the two main players of the regeneration process: myogenic progenitors (MPs) and fibro/adipogenic progenitors (FAPs) [5,6,8,51,52]. Our single-cell analysis revealed that both cell types more than doubled in number from day 3 to 10 (Figure 3D,E).

We could not obtain mass cytometry grade antibodies, specifically recognizing the paired box protein PAX7 antigen, which labels satellite cells. Thus, we resorted to using the α7-integrin antigen as a marker of the myogenic progenitor (MP) cluster (Figure 3D), including both satellite cells and myoblasts. Within the population expressing α7-integrin, we could identify two smaller clusters expressing different levels of vimentin (Figure S1C). During regeneration, the MP cell population, after an initial decrease, became more populated (Figure 3D). The relative abundance of the two subpopulations changed in time with the vimentin expressing MP, significantly increasing in number at day 3 (Figure S1C). At day 3 post-injury, the MPs accounted for about 6% of the total recorded cell events.

The FAP compartment was defined in our t-SNE map by a cluster of cells expressing SCA1, CD34, CD140α, CD90.2, and vimentin. Similar to the MP population, albeit with a different trend, the FAP population became more numerous as the regeneration process progressed, reaching the maximum expansion between 5–10 days after CTX injury (about 11.3% of total events), to return to almost control levels at day 20 (Figure 3E).

Our analysis also allowed us to characterize the kinetic of vessel-associated populations during regeneration [53,54]. These were identified, among CD45 negative cells, as they expressed the CD31 antigen. We were able to distinguish two subpopulations of cells expressing additional markers of endothelial populations at different levels (SCA1, CD146, and CD34) (Figure S1D). CD31 cluster 4 was considered a myoendothelial cell subpopulation, containing cells that also express high levels of the α7-integrin antigen [55]. Overall, we observed that the whole endothelial progenitor pool followed a kinetic that was different from the populations examined so far. They decreased significantly in number in the first few days after damage and then increased over the homeostatic level toward the end of the regeneration process (Figure 3F).

We also considered a population of cells whose expression profile was reminiscent of that of pericytes [56,57]. They were characterized by the expression of CD146, CD140β, and α7-integrin (Figure 3G). These pericyte-like cells followed a trend that was very similar to that of endothelial cells, with a sharp decrease early after damage and a rapid increase in the late regeneration phase, when vascularization took place (Figure 3G). We also looked at a cluster of cells that we were not able to match to any of the cell types described so far. We dubbed this population as mesenchymal-like cells, as they were highly positive for CD90.2 and CD140β markers. The abundance of this population followed a kinetic that seemed to be governed by the regeneration process and was also very similar to that of the classical endothelial cells (Figure 3H).

### 3.3. Single-Cell Profiling of the mdx Muscle

**Figure 4:**
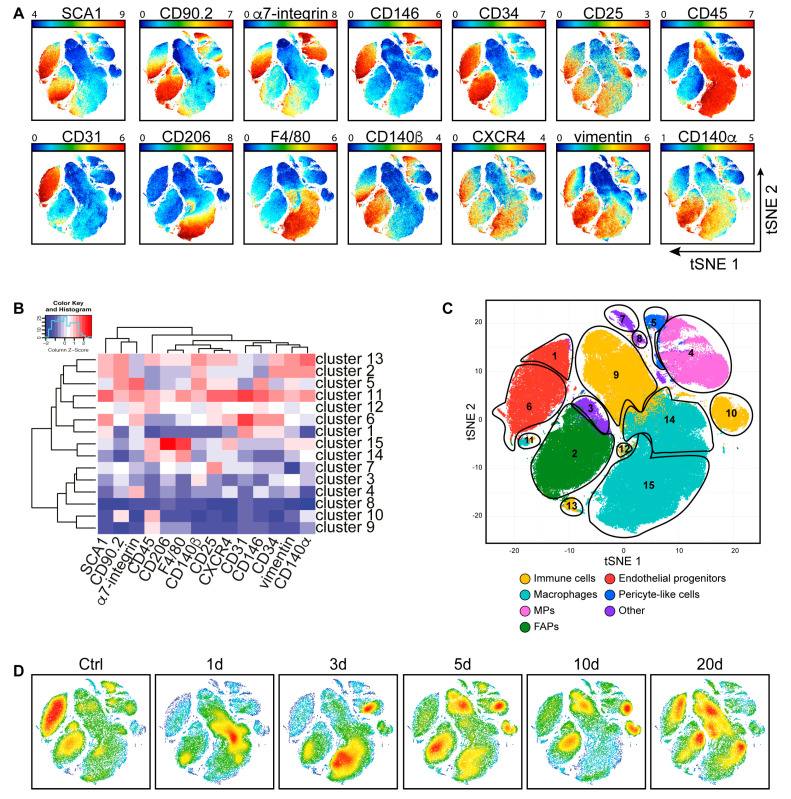
Identification of the main mdx skeletal muscle cell populations upon CTX-induced injury. (**A**) A mononuclear cell suspension was purified from uninjured and CTX-injured mdx hind limb muscles. The different samples were combined, barcoded, and analyzed to create a single t-SNE map colored according to SCA1, CD90.2, α7-integrin, CD146, CD34, CD25, CD45, CD31, CD206, F4/80, CD140β, CXCR4, vimentin, and CD140α expression levels (blue: low expression; high expression of the selected marker). (**B**) FlowSOM heatmap of column normalized (Z-score) marker expression for each of the 15 clusters identified by Cytofkit analysis. Colors varied according to the expression level of each considered marker in a blue to red scale, indicating low and high expression, respectively. (**C**) Cell population clusters defined by overlapping the expression for each of the different analyzed antigens were projected onto t-SNE space and assigned to specific colors (yellow: immune cells; light blue: macrophages; pink: myogenic progenitors (MPs); dark green: fibro/adipogenic progenitors (FAPs); red: endothelial progenitors; blue: pericyte-like cells; purple: other). (**D**) Density plots colored by density (blue: low density; red: high density), showing cell abundance variations at different times during the regeneration process.

We further aimed at characterizing the response to acute damage and the ensuing regeneration process in the mdx dystrophic muscle. Mononuclear cells were isolated from the uninjured and CTX-injured skeletal muscles of mdx mice, following the same procedure described for the wt in the previous section (Figure 4A–D). Mononuclear cells were separated from muscle fibers, barcoded, labeled with the same antibody panel, and prepared for mass cytometry. The resulting single-cell antigen expression profile was processed, as described for the wt cells, in order to obtain a two-dimensional representation of antigen expression in the different cell types. As the wt and mdx mass cytometry analyses were performed at different times, the resulting t-SNE maps could not be directly compared (Figure 2A and Figure 4A). However, by comparing the antigen expression profiles in the different clusters, we were able to associate each cluster to one of the main muscle mononuclear cell types and match it to those observed in the wt (Figure 4B,C). As observed in injured-recovering wt muscles, the relative numerosity of the different cell populations also changed after damage and during regeneration in the mdx model (Figure 4D).

**Figure 5:**
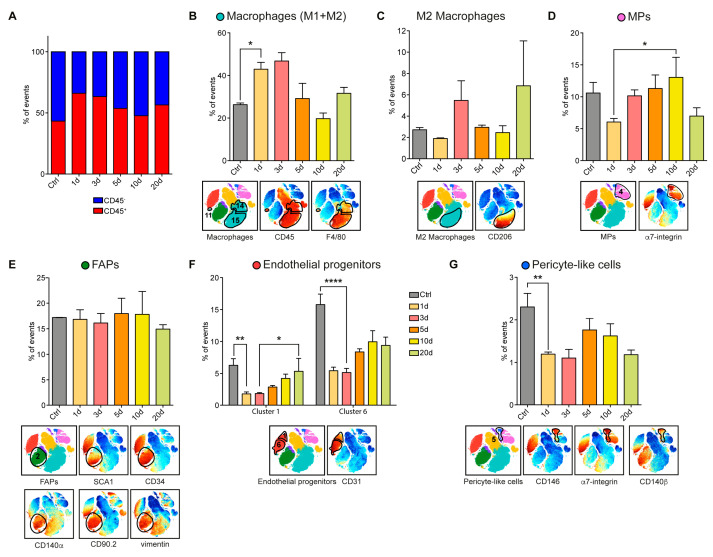
Characterization of the population dynamics induced by CTX injury in the mdx skeletal muscle at day 1, 3, 5, 10, 20 after injury. (**A**) Bar plots, showing the CD45^-^ (blue) / CD45^+^ (red) ratio in the mdx mononuclear cells at different time points after CTX injury. Data were represented as mean, and the statistical significance was estimated by two-way ANOVA. (**B**) t-SNE maps, showing the population gated as macrophage, colored for CD45 and F4/80 expression (red: high expression; blue: low expression) together with the macrophage trend observed in uninjured and CTX-injured mdx hind limb muscles. (**C**) M2 macrophages, expressing the CD206 antigen. (**D**) t-SNE maps of the myogenic progenitor (MP) population colored according to the levels of α7-integrin expression (red: high expression) and MP population dynamics during regeneration. (**E**) t-SNE maps, representing fibro/adipogenic progenitors (FAPs) identified among uninjured and CTX-injured mdx hind limb muscles, colored according to the expression of SCA1, CD34, CD140α, CD90.2, and vimentin (red: high expression; blue: low expression). The bar plot illustrated the population dynamics during regeneration. (**F**) Bar plots, showing the abundance of two different subclusters (cluster 1 and cluster 6) of endothelial progenitor cells at different time points. (**G**) t-SNE maps of pericyte-like cells identified in uninjured and CTX-injured mdx hind limb cell populations. Cell clusters were colored according to the expression of CD146, α7-integrin, and CD140β (red: high expression; blue: low expression). The bar plot illustrated the population dynamics during regeneration. Population abundance was assessed by calculating the percentage of cells in any given population over the total number of mononuclear cells in each sample (n = 3). The statistical significance was estimated by one-way ANOVA. All data were represented as mean ± SEM, and the statistical significance was defined as * *p* < 0.05; ** *p* < 0.01; **** *p* < 0.001.

The number of cells in the CD45^+^ compartment (clusters 9, 10, 11, 12, 13, 14, and 15), as a whole, did not change significantly along the regeneration process (Figure 5A). However, the distribution of cells in the different populations in the compartment was found to be highly dynamic. In particular, macrophages (clusters 11, 14, and 15), which were already abundant in the mdx muscle before acute damage, increased significantly in the early days after CTX treatment, as observed in the wt model, to return to mdx baseline levels, approximately 30% of total mononuclear cells, at day 20 (Figure 5B). M2 macrophages, on the other hand, remained rather constant early after an injury to increase only late in the regeneration process (Figure 5C).

When compared to wt, myogenic progenitors (MPs) (cluster 4) and fibro/adipogenic progenitors (FAPs) (cluster 2) followed a different trend. As the mdx muscle was under chronic stress, we observed that both progenitor cell populations were more populated in the absence of acute damage. However, while MPs decreased in number as early as one day after acute damage to return to unperturbed levels at later times, FAPs remained at a constant high level along the whole regeneration process (Figure 5D,E).

As already observed for the wt, also for the mdx mouse model, the CD31 expressing cells (clusters 1 and 6), defining endothelial progenitors, comprised two sub-populations differing for the expression of α7-integrin and other markers (CD90.2, CD140β, and CXCR4) (Figure S1E). The population expressing higher levels of α7-integrin was twice as abundant as the other. Nevertheless, both sub-populations reacted similarly to acute damage and first dropped in abundance by a factor of approximately three, to recover at the late stages of the regeneration process (Figure 5F). However, consistently, the cluster enriched in α7-integrin expressing cells (cluster 6) had not fully recovered at day 20, suggesting that the vascularization was still ongoing at times when histology seemed to indicate completion of the regeneration process. This differed from what was observed in the wt. Consistent with this consideration, and differently from what observed in the wt, also the pericyte-like population (cluster 5) behaved similarly, dropping in abundance immediately after damage and then slowly recovering without, however, reaching full recovery at day 20 (Figure 5G).

## 4. Discussion

The skeletal muscle has a remarkable capacity to self-repair if damaged [7,8,9,10,49,58]. However, this healing process may fail, owing to excessive damage, aging, or genetic disorders [12,59,60,61,62]. As a consequence of this failure in the repair process, as in muscle dystrophies, the tissue undergoes degeneration, leading to progressive muscle wasting and weakness characterized by chronic inflammation and, at later stages, fat and fibrotic tissue infiltrations [9,13,14].

Here, we exploited the resolution power of mass cytometry to characterize the modulation of the profile of the mononuclear cell population following chronic or acute damage [63,64,65]. To this end, we assembled a panel of 23 metal-tagged antibodies and characterized, at the single-cell level, the dynamics of muscle mononuclear cell populations after acute damage in wild type (wt) and in a mouse model of Duchenne muscular dystrophy (mdx). The regeneration process was monitored by examining samples of muscle mononuclear cells at 1, 3, 5, 10, and 20 days after cardiotoxin injection in wt or mdx mice [29,30].

This approach yielded a reach multiparametric dataset, disclosing the details of how the composition and heterogeneity of mononuclear cell populations changed in time as the muscle healing process proceeded. By applying a dimensionality reduction technique, such as the t-distributed stochastic neighbor embedding (t-SNE) algorithm, we generated bidimensional maps, providing a visual description of the regeneration process. Furthermore, this representation contributed to revealing subtle differences in the expression of specific markers in subpopulations within the major clusters that identified “classical” muscle populations. This population heterogeneity could not be only explained by experimental variability, even if any functional implication in muscle physiology or pathology remains to be established. In this report, we only dwelt upon a few of these t-SNE map features, while the dataset remains as a resource for additional analysis for the community.

A time-dependent variation in the abundance of muscle mononuclear cells was observed in wt mice, starting at day 1, indicating that the system sensed the damage and promptly responded to restore muscle tissue homeostasis. Inflammatory cells were the predominant population 3 days after the injury, accounting for over 40% of the total mononuclear cells. The activation of the inflammatory compartment was limited to the first few days after the injury as macrophages returned to baseline levels after 10 days.

Fibro/adipogenic progenitors (FAPs), here identified as SCA1^+^, CD34^+^, CD140α^+^, CD90.2^+^, and vimentin^+^ [66], stimulate satellite cell activation and differentiation, thus playing a positive role in muscle regeneration [8]. FAPs are quiescent in intact muscles, while they proliferate in response to injury [8,67,68]. Consistently, we observed that FAPs, after CTX injection, rapidly expanded, in wt muscles, reaching a peak between days 5 and 10, to return eventually to baseline level, having accomplished their function of providing a transient production of pro-differentiation signals and of depositing extracellular matrix for muscle remodeling.

In response to the secreted inflammatory and fibro/adipogenic stimulating signals, the myogenic compartment also promptly activated at day 3, as confirmed by vimentin expression, which marked activated MPs or myoblasts [69]. After an initial drop in concentration, α7-integrin^+^ MPs, including satellite cells and myoblasts, expanded on day 3, still remaining high on day 10, when they started to decrease.

Different kinetics was observed for endothelial progenitor cells, here, characterized as CD31, SCA1, CD34, and CD146 expressing cells, and for pericyte-like cells, mainly identified as CD146 and CD140β-positive cells. The endothelial and pericyte-like cell contribution to skeletal muscle recovery only took place at the end of the regeneration process; once the inflammatory cells were removed, FAPs decreased in number, and MPs differentiated.

Due to the incomplete nature of our panel, some cell clusters in our t-SNE maps remained loosely defined. One cluster included cells with an antigen repertoire reminiscent of mesenchymal cells, as they were positive for the mesenchymal markers CD90.2 and CD140β [70]. This population had a clear kinetic, suggesting a late intervention of the mesenchymal population in the muscle regeneration process.

We also investigated the response to acute injury of a muscle environment that was already chronically perturbed as in the mdx mice. Mononuclear cells extracted from the muscle of mdx mice did not experience any significant increase in number following cardiotoxin injury. However, by looking into the details of the population profiles at each time point, we observed a significant modulation of the population distributions as the increase in the number of the cells in one population was counterbalanced by the decrease in another one. For instance, while we observed that the cells in the inflammatory compartment, essentially macrophages, expanded early after damage, the endothelial and pericyte-like clusters dropped in numerosity to recover the initial values only late in the process. On the other hand, differently from wt, the myogenic and fibro/adipogenic compartments, the two main players in muscle regeneration, showed little variation during the regeneration process, probably because they were already chronically activated.

Overall, our multiparametric analysis offered a comprehensive description of both muscle tissue homeostasis and the rearrangements induced in the mononuclear cell population profile by a perturbation of the muscle system, be it a chronic condition, as in the case of mdx mice, or acute stress, as that triggered by cardiotoxin injection.

## Figures and Tables

**Table 1:** List of the metal-tagged antibodies used in the mass cytometry experiments.

Antibody	Metal
Anti-mouse CD45	147Sm
Anti-mouse Ly-6A/E (SCA1)	164Dy
Anti-mouse CD90.2 (Thy-1.2)	156Gd
Anti-mouse CD146	141Pr
Anti-mouse F4/80	146Nd
Anti-mouse CD140α	148Nd
Anti-mouse CD140β	151Eu
Anti-mouse α7-integrin	161Dy
Anti-mouse CD206	169Tm
Anti-mouse CD34	144Nd
Anti-mouse CXCR4	159Tb
Anti-mouse CD4	172Yb
Anti-mouse CD25 (IL-2R)	150Nd
Anti-vimentin	154Sm
Anti-CD31 (PECAM-1)	165Ho
Anti-pan-actin	175Lu
Anti-mouse interleukin-6 (IL-6)	167Er
Anti-phospho-Akt (S473)	152Sm
Anti-phospho-Stat1 (Y701)	153Eu
Anti-phospho-Erk1/2 (T202/Y204)	171Yb
Anti-phospho-Stat3 (Y705)	158Gd
Anti-cleaved caspase3	142Nd
Anti-phospho-Creb	176Yb

CD: Cluster Differentiation; SCA1: Stem Cell Antigen1; CXCR4: C-X-C Motif Chemokine Receptor 4; PECAM-1: Platelet Endothelial Cell adhesion-1; Akt: RAC alpha serine/threonine-protein kinase; Stat1: Signal Transducer and activator of transcription 1; Erk1/2: Extracellular signal-regulated kinases 1, 2; Stat3: Signal transducer and activator of transcription 3; cleaved caspase3: **c**ysteine-aspartic proteases; Creb: cAMP response element-binding protein.

## Supplementary Materials

The following are available online at https://www.mdpi.com/2073-4409/9/7/1723/s1. Figures S1–S4 and “FCS folder” with two subfolders (“MDX_FCS” and “WT_FCS”) containing the mass cytometry raw data (.fcs files).
