# Supplementary material for: High-Dimensional Single-Cell Quantitative Profiling of Skeletal Muscle Cell Population Dynamics during Regeneration
Source: Cells. 2020 Jul 18;9(7):1723. doi: 10.3390/cells9071723 (PMC7407527; doi:10.3390/cells9071723)
Supplement: Supplementary file 1 [file cells-09-01723-s001.pdf]

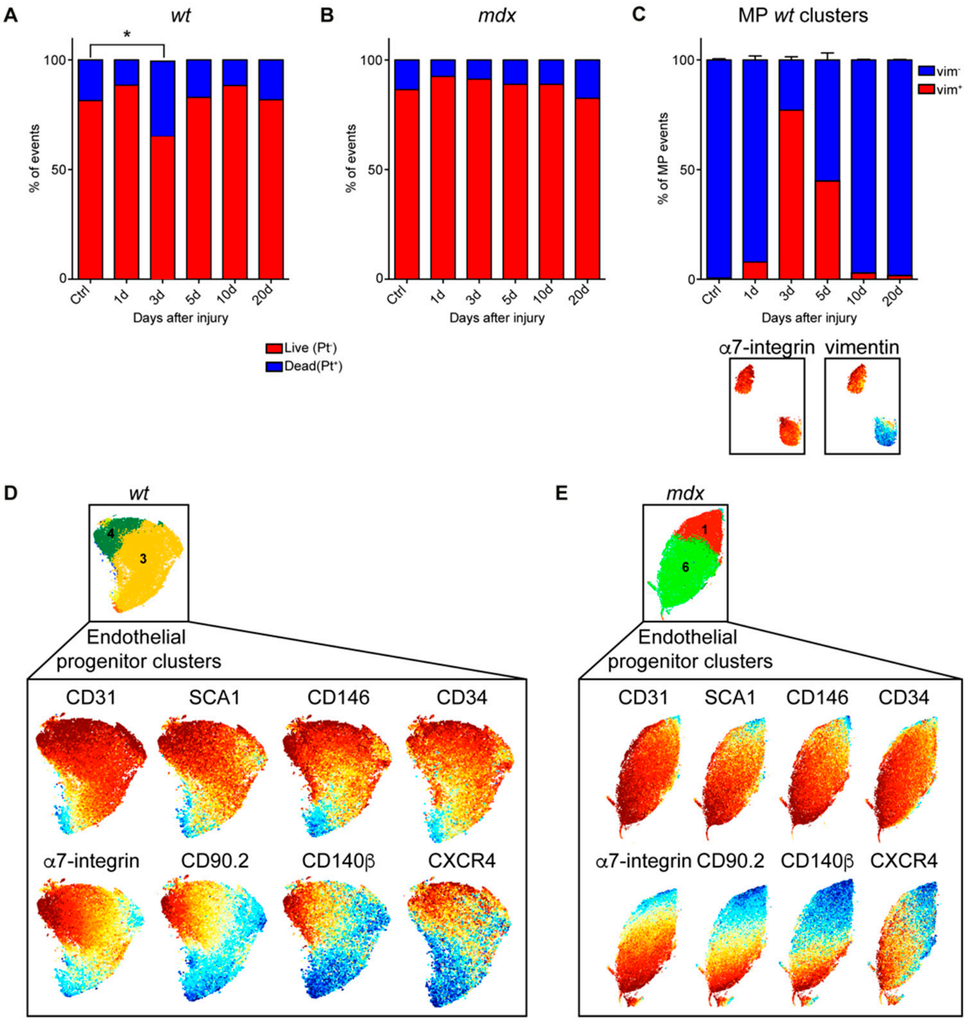

Supplementary Figure 1. (**A, B**) Bar plots showing the relative abundance of live (Pt$^-$, red) and dead (Pt$^+$, blue) cells identified on singlets at different time points after CTX injury in wt (**A**) and mdx (**B**) skeletal muscle tissues. (**C**) t-SNE map of the Myogenic Progenitor (MP) subclusters characterized by different expression levels of vimentin (red, high expression; blue, low expression) in the wild type muscle. Bar plots showing the relative abundance of the vimentin$^-$ (blue) and vimentin$^+$ (red) subpopulations in the MP clusters at different time points after CTX injury. Data are represented as mean. (**D, E**) t-SNE maps of mononuclear cell populations showing two subclusters of CD31$^+$ endothelial progenitor cells in the wt (**D**) and mdx (**E**) skeletal muscles. The different t-SNE are color coded according to the expression of surface antigens that characterize the identified cell types (CD31, SCA1, CD146, CD34, α7-integrin, CD90.2, CD140β and CXCR4). The statistical significance was estimated by Two-way ANOVA. All data are represented as mean ± SEM and the statistical significance is defined as * $p < 0.05$; ** $p < 0.01$; *** $p < 0.001$; **** $p < 0.0001$.

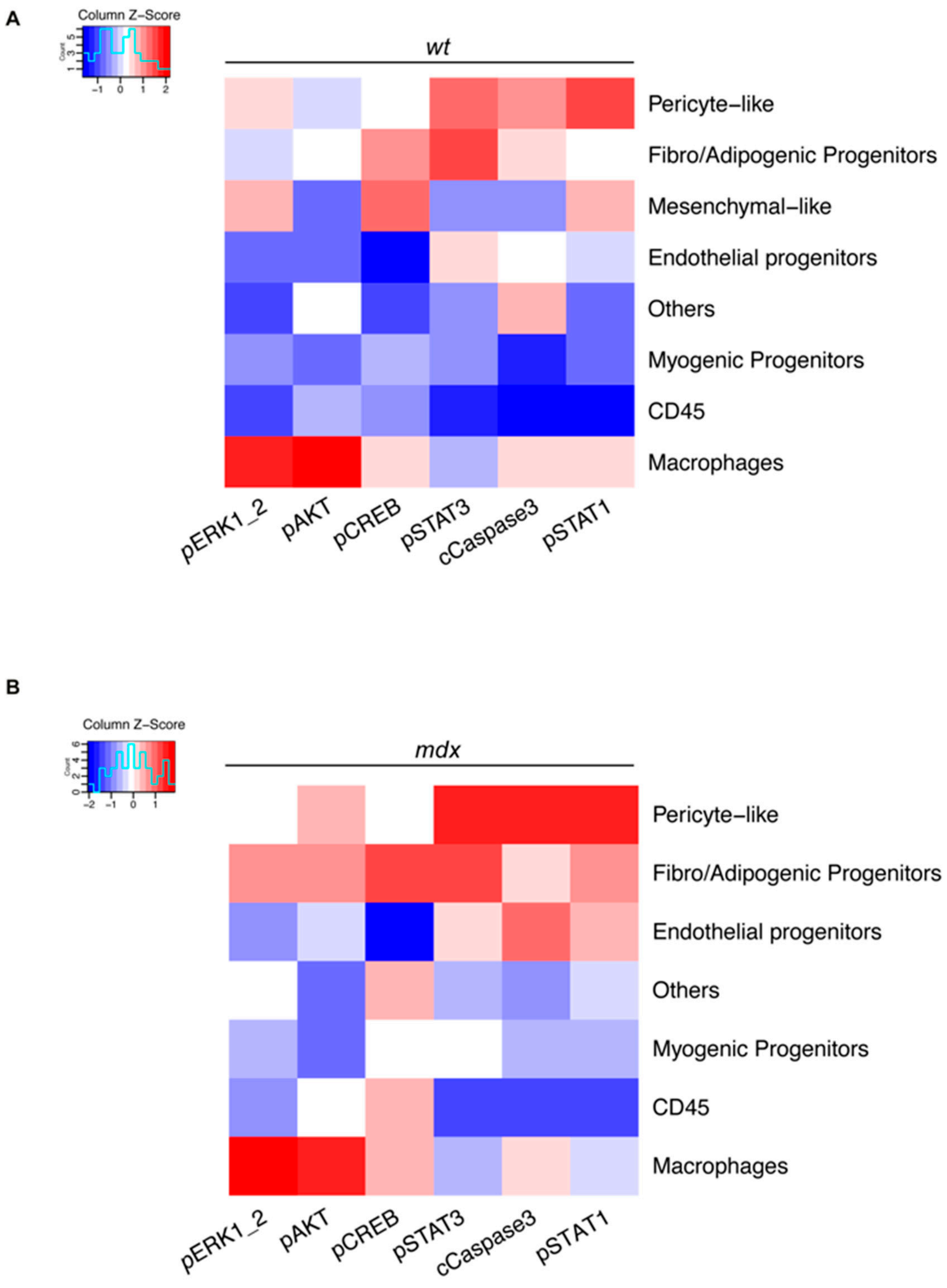

Supplementary Figure 2. (A, B) Heatmaps representing the mean intensity of intracellular marker expression in the different subpopulations identified in the whole wt **(A)** and mdx **(B)** dynamics experiments. Data are scaled by column and represented in a blue-red scale from lower to higher expression.

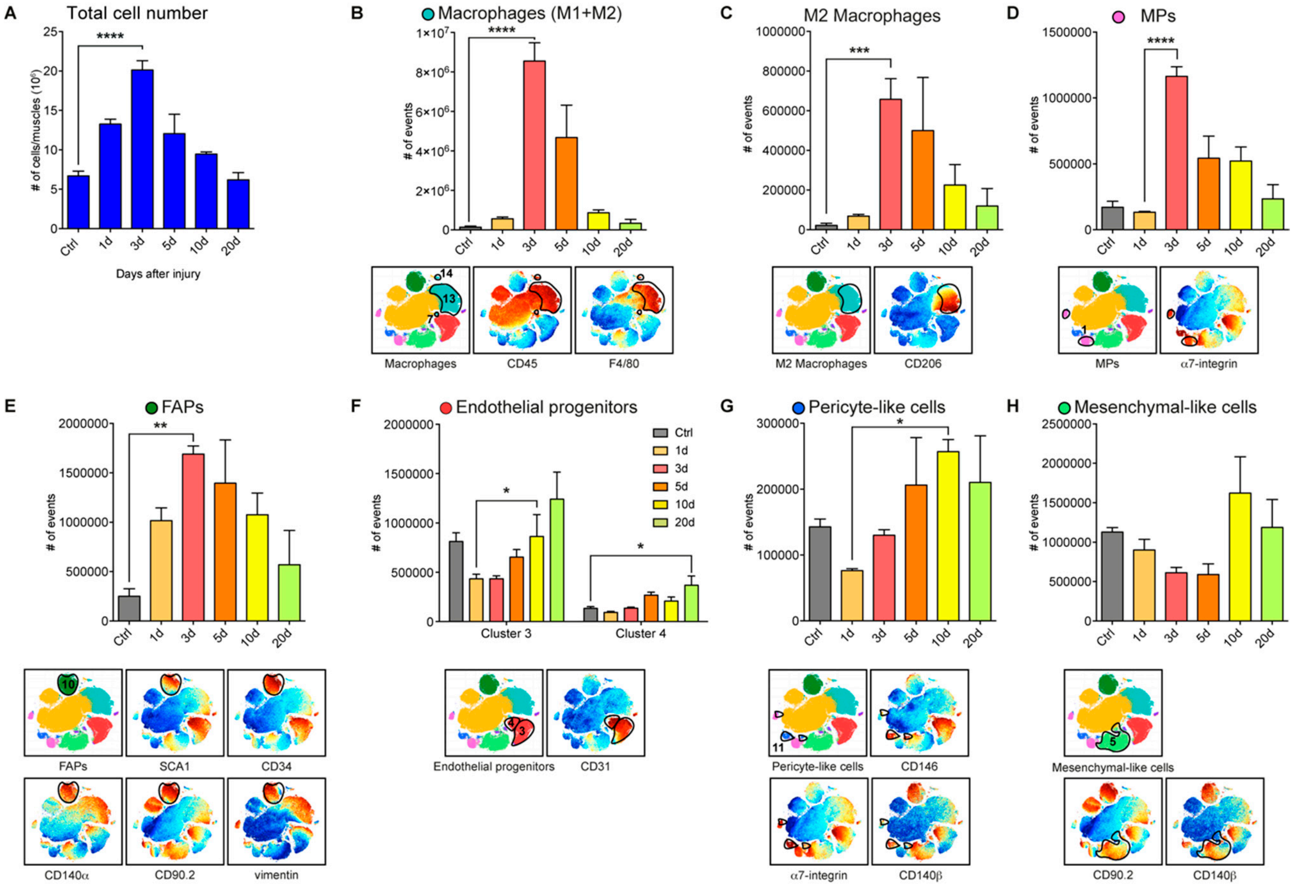

Supplementary Figure 3. (A) Number of cells (in millions) extracted from uninjured and CTX-injured wt mice (n=3, for 5d time point n=2). All data are represented as mean ± SEM and the statistical significance was estimated by Two-way ANOVA. **(B-H)** Identification in the t-SNE maps of mononuclear cell populations. The different plots are color coded according to the expression of surface antigens that characterize the relevant cell types. The bar plots quantitate the cell number in the wt limb muscles at different times during regeneration. Cell numbers were assessed on the total number of cells in each sample as in A (n=3, for 5d time point n=2). The statistical significance was estimated by One-way ANOVA. All data are represented as mean ± SEM and the statistical significance is defined as * $p < 0.05$; ** $p < 0.01$; *** $p < 0.001$; **** $p < 0.0001$.

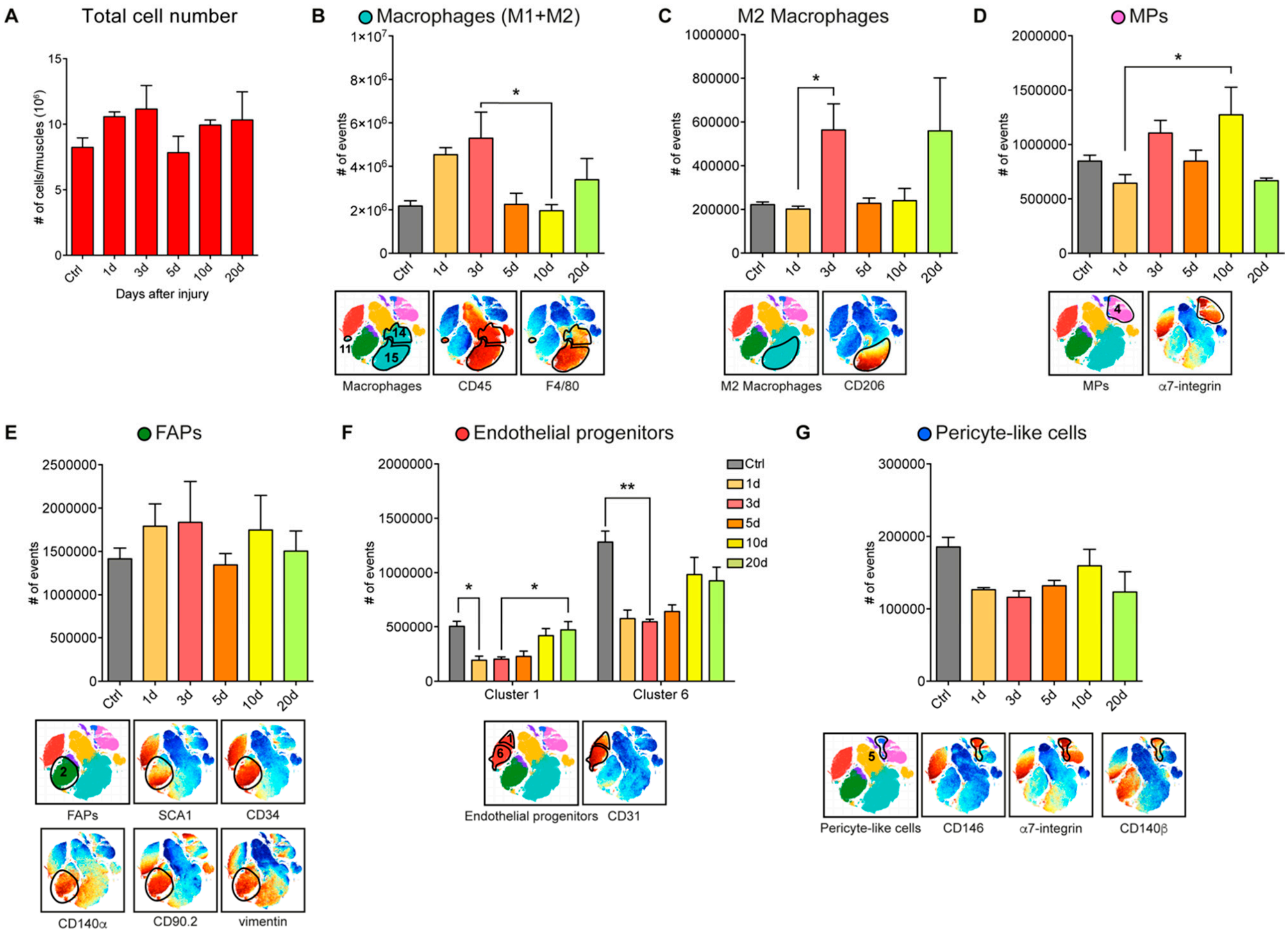

Supplementary Figure 4. (A) Number of cells (in millions) extracted from uninjured and CTX-injured mdx mice (n=3). All data are represented as mean ± SEM and the statistical significance was estimated by Two-way ANOVA. **(B-G)** Identification in the t-SNE maps of mononuclear cell populations. The different plots are color coded according to the expression of surface antigens that characterize the relevant cell types. The bar plots quantitate the cell number in the mdx limb muscles at different times during regeneration. Cell numbers were assessed on the total number of cells in each sample as in A (n=3). The statistical significance was estimated by One-way ANOVA. All data are represented as mean ± SEM and the statistical significance is defined as * $p < 0.05$; ** $p < 0.01$; *** $p < 0.001$; **** $p < 0.0001$.
